# The Intentional and Unintentional Human Food Subsidy Landscape for a Large Carnivore

**DOI:** 10.1002/ece3.71853

**Published:** 2025-08-21

**Authors:** Matthew M. Smith, David M. MacFarland, Jennifer L. Price Tack, Jonathan N. Pauli

**Affiliations:** ^1^ Department of Forest and Wildlife Ecology University of Wisconsin Madison Wisconsin USA; ^2^ Wisconsin Department of Natural Resources Office of Applied Science Rhinelander Wisconsin USA

**Keywords:** American black bear, diet, foraging, human‐wildlife interactions, stable isotopes, *Ursus americanus*

## Abstract

Humans have transformed ecosystems and resource availability. Many species exploit these novel resources, which can increase conflict between humans and wildlife. This is particularly true for large carnivores that readily consume human foods, which can lead to conflict. However, disentangling the different forms of human food subsidies, their drivers across a landscape, and potential consequences for conflict has not been explored. American black bears ( *Ursus americanus* ) consume large quantities of unintentional (e.g., refuse, crops) and intentional human food (e.g., hunting bait). Up to 40% of a wild bear's diet can be from human food subsidies. This consumption has been associated with increasing numbers and more conflict. The state of Wisconsin, USA, has a liberal bear baiting policy and high densities of bears compared to neighboring states and has the potential for high consumption of human foods and conflict. We estimated the proportional diets of black bears using stable isotope analysis (δ^13^C and δ^15^N) from a statewide sampling in Wisconsin. We modeled the response of diets to landscape variables likely to influence the consumption of either natural food items and two sources of human food subsidies: intentional and unintentional. We then predicted a human subsidy landscape that explored the spatial variation between intentional and unintentional sources and modeled the relationship between human food subsidies and conflict. We found substantial consumption of intentional (7%; 95% CI [0%, 25%]) and unintentional (32%; 95% CI [27%, 38%]) subsidies. The consumption of intentional subsidies increased in areas with public lands and higher hunter activity, while the consumption of unintentional subsidies increased with corn production and less natural land cover. We found the number of reported complaints increased with the consumption of unintentional human subsidies. Our predicted map of human subsidy consumption disentangled the form of the human food subsidy and showed that the consumption of unintentional human food subsidies along their expanding range can lead to greater conflict with people and property. Our mapped subsidy landscape can be used by managers to target management actions to reduce the availability of human subsidies and to predict areas of human‐wildlife interactions.

## Graphical abstract

Our findings highlight important differences between the source of human subsidy and how it enters the natural system and leads to human‐wildlife conflict. Ultimately, we can target management actions that influence habitat that can mediate human subsidy consumption and identify areas to implement strategies that promote safe human‐wildlife interactions.

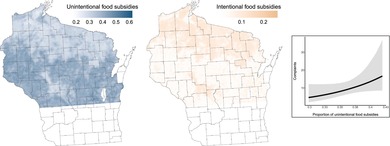

## 1 Introduction

Human activities have transformed ecosystems globally and are the main driver of environmental change (Kennedy et al. 2019; Vitousek et al. 1997). In particular, land use change has altered > 70% of terrestrial ecosystems and pushed wildlife into human‐modified landscapes where natural land cover gives way or becomes interspersed among anthropic cover (e.g., agriculture and urban areas) (Ellis et al. 2013; Foley et al. 2005). The loss of natural land cover and increasing human‐modified landscapes have resulted in increased interactions between humans and wildlife (Woodroffe and Ginsberg 1998). For many species, human impacts through direct persecution and resource loss have resulted in population declines and range contractions (e.g., Laliberte and Ripple 2004; Cardillo et al. 2005). Despite initial declines, some species are recovering to portions of their historical range and even expanding into new ones; however, recovery now occurs in landscapes that have been fundamentally altered by human impact (Hobbs et al. 2014). In particular, animals living within or close to human‐modified landscapes typically experience a change in resource availability, especially food subsidies.

The ability to exploit human‐modified landscapes often hinges on a species' ability to make use of added food resources (Manlick and Pauli 2020). Such human food subsidies are often energy rich, abundant, and highly predictable in space and time; thus, making their exploitation a rewarding foraging strategy (Fehlmann et al. 2021; Oro et al. 2013). The high predictability in space and time of human food subsidies can make forage easier to find and consume compared to natural sources, and when abundant, can often improve nutritional condition (i.e., body condition and body mass) (Nelson et al. 2024). Human food subsidies can have direct effects on individual fitness (Oro et al. 2008), behavior (Yirga et al. 2012; Newsome et al. 2015), and space use (Brunk et al. 2021; Newsome et al. 2013) with cascading effects on populations including abundance and distributions (Fedriani et al. 2001). Indeed, a number of taxa have become adept at exploiting novel food resources in human‐modified landscapes, including a diverse array of both mammalian and avian species, with effects on their abundances and distributions (Carey et al. 2012). However, even though some species benefit from these contributions, human food consumption can alter natural community composition, interactions, and pathogen spread (Oro et al. 2013). From an individual perspective, the exploitation of these areas can also come at a cost, leading to increased conflict with humans (Moss et al. 2016), altered physiology (Schulte‐Hostedde et al. 2018), and disease transmissions (Hassell et al. 2017). Thus, the alteration of a species' diet through human food subsidies is often not overtly apparent or easily quantified but can have extensive consequences for ecosystem function and human‐wildlife interactions.

Human food subsidies can be intentionally provided to wildlife through supplemental feeding to support at‐risk populations (López‐Bao et al. 2008; Ewen et al. 2015), reduce human‐wildlife conflict (Kubasiewicz et al. 2016), or enhance the viewing of wildlife (e.g., bird feeders) and hunting by increasing success and selectivity through baiting (Putman and Staines 2004). Alternatively, human food subsidies may be unintentional through food production, crop residuals, and refuse (Oro et al. 2013). Both intentional and unintentional food subsidies contribute billions of tons of food resources per year to ecosystems globally (Kaza et al. 2018; Oro et al. 2013). While the sources of human food subsidies are well documented, the landscape features influencing consumption have received little attention, making it necessary to identify where human food subsidies are entering the system to help predict conflict and create opportunities for management.

American black bears ( *Ursus americanus* ) are opportunistic omnivores that can exploit a variety of different foods including those provided by humans (Lewis et al. 2015). Bears can move between wildlands and urban areas to forage and meet energetic needs (Lewis et al. 2015; Merkle et al. 2013). Indeed, black bears will alter their behavior to exploit human food subsidies including long‐range movements and shift daily activity to avoid humans while still accessing anthropogenic food resources (Beckmann and Berger 2003a; Breck et al. 2009; Zeller et al. 2019). In addition to behavioral changes, landscape composition including human density and proportion of agricultural land has been positively related to increased consumption of human food subsidies for bears (Ditmer et al. 2016; Kirby et al. 2016). While bear diets have been shown to track landscape features and availability, the landscape drivers and different ‘diet landscapes’ between the foraging of intentional and unintentional human food subsidies have not been explored.

Globally, the intentional feeding of bears is widely practiced to enhance hunting opportunities, viewing, and as a conflict‐mitigation strategy (i.e., diversionary feeding) (Garshelis et al. 2017; Kavčič et al. 2015; Kirby et al. 2017). In North America, baiting bears to harvest is common and allowed in nine U.S. states and seven Canadian provinces. Regulations for bear baiting vary but are generally restricted by the number of baits or the days deployed. The state of Wisconsin, USA is unique in that it has liberal baiting regulations that extend 145 days prior to the 35‐day hunting season, from mid‐April through early October that mirrors the entire active (i.e., non‐hibernation) period for bears. Intentional baiting is restricted to non‐animal products, and is generally high‐sugar foods (e.g., trail mix, cookies, donuts, and cereals), and in northern Wisconsin, black bears may encounter dozens of bait piles per year (female: 4–13 baits/year; male: 21–63 baits/year) and can constitute a high proportion (> 40%) of individual diets (Kirby et al. 2017). During the period of black bear expansion, agriculture production has increased, and in particular, corn production has increased threefold (USDA Economic Research Service). While most corn production occurs outside of the core range, the southerly expansion of black bears has increased their occurrence into regions dominated by agricultural land cover. These factors can lead bears into areas where more conflict can occur and potentially lead to an ecological trap if crop production changes. Thus, identifying variation in the human subsidy landscape can identify the landscape features that may mediate or enhance the consumption of human food subsidies to better anticipate use across an expanding range and the potential for human‐wildlife interactions.

We explored differences in assimilated diets of black bears and the effect of landscape characteristics on the consumption of multiple diet items through a statewide sampling effort. We sought to disentangle the consumption of two sources of human food subsidies: intentional (i.e., hunting bait) and unintentional (i.e., agriculture and refuse) and by doing so identify spatial variation in human food subsidies, help predict conflict, and understand where opportunities for management may be most beneficial. We estimated proportional diets, the proportion of each diet item that contributes to the total diet, of black bears and their foods using stable isotope analysis (δ^13^C and δ^15^N) to better understand the landscape of human subsidies across their distribution in Wisconsin. We modeled the response of proportional diets to a suite of landscape variables that represented composition, configuration, and productivity that were likely to influence the consumption of either natural food items or human food subsidies. We predicted that proportional diets would be positively associated with changes in the productivity and availability of both natural and human‐derived resources. Then, to explore the relationship between human food subsidies and conflict, we spatially predicted the consumption of human food subsidies across their distribution and modeled the response to reported complaints. Our work aims to better understand the landscape conditions likely to lead to greater consumption of human food subsidies and disentangle the specific proportional contributions from two contrasting sources of human food to anticipate human‐wildlife interactions of an expanding large carnivore population.

## 2 Materials and Methods

### 2.1 Sample Collection

**FIGURE 1:**
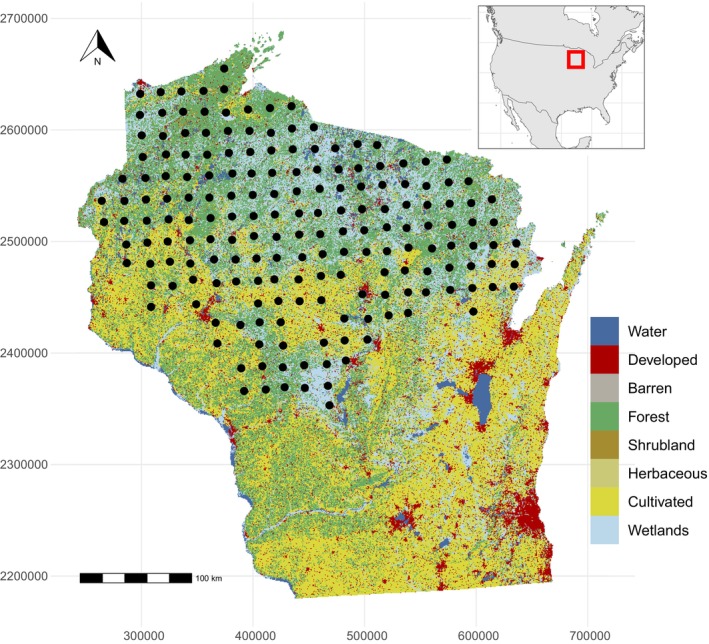
Sampling locations of American black bear ( *Ursus americanus* ) hair (black dots) in Wisconsin and land cover from the 2019 National Land Cover Database. Samples were collected from a noninvasive hair trapping grid during May and June of 2019.

We collected hair from black bears in May and June 2019 from a noninvasive sampling grid comprised of 844 barbed wire hair corrals clustered in 2 × 2 groups across their distribution in Wisconsin (Figure 1). Corral groups were separated on average by 1711 m. Each corral was baited with peanut butter and oil slurry and liquid smoke scent lures. Corrals were checked once a week for four consecutive weeks to collect hair samples from each barb. Samples from black bears and diet items were collected for stable isotope analysis of δ^13^C and δ^15^N (Table S1). We prepared samples for isotopic analysis by rinsing 3× with a 2:1 chloroform: methanol solution to remove surface contaminants, homogenized, and dried at 56°C for 72 h or until dry (Pauli et al. 2009). Analysis of stable isotopes was conducted at UC‐Davis Stable Isotope Facility using a PDZ Europa ANCA‐GSL elemental analyzer interfaced to a PDZ Europa 20–20 isotope ratio mass spectrometer (Sercon Ltd., Cheshire, UK). Isotope ratios are reported in delta (δ) notation in per mille (‰) relative to the international standards Vienna Peedee Belemnite and atmospheric nitrogen. Samples were collected in the late spring and early summer of 2019 and, thus, represented assimilated diet from the previous year (Hopkins et al. 2014). We sampled all primary diet groups including both natural forage and human‐sourced foods. Natural forage included known diet items of vegetation (soft mast, hard mast, and herbaceous plants) and animal matter (deer and ants) from previously published literature in Wisconsin (Kirby et al. 2017). To increase sample size and spatial distribution of diet items in Wisconsin, we supplemented hard mast and deer with additional samples collected in 2022. We used published isotopic values for human‐sourced foods that included bait (Kirby et al. 2017), food waste (Newsome et al. 2015), and corn (Ditmer et al. 2016). The isotopic values of bait were calculated from 42 bait stations in northern Wisconsin from a mixture of items found at each station including trail mix, donuts, cookies, candies, cereals, and frosting (Kirby et al. 2017). We included diet sources outside of the primary year of the study; however, substantial annual changes in bulk isotopic signatures are rarely observed (Ben‐David and Flaherty 2012).

### 2.2 Landscape Variables

To calculate landscape conditions around sampling locations, we extracted variables within a 54 km^2^ buffer that represents the average home range of male and female black bears in Wisconsin (Massopust 1984; Kessler 1994). We selected a set of landscape variables a priori representing landscape composition, configuration, and productivity that could influence the consumption of human‐derived subsidies by black bears. We derived metrics of landscape composition and configuration from the National Land Cover Dataset (NLCD; Figure 1) from the closest available year (2019) using the R (R Core Team 2024) package *landscapemetrics* (Hesselbarth et al. 2019). We created a natural land cover category by combining NLCD cover classes (forest, shrubland, herbaceous, and wetlands) and calculated the proportion, edge density, and connectivity (contiguity index) around each sample location (Cushman et al. 2008). We used a dynamic habitat index to represent vegetation productivity over the course of the year as a proxy for resource availability (Hobi et al. 2017; Radeloff et al. 2019). Specifically, we calculated cumulative productivity in 2018 derived from the fraction absorbed photosynthetically active radiation, a product that combines MODIS and VIIRS sensors (48 images; Pu et al. 2024). We also incorporated specific predictors that could influence the availability of human food waste subsidies using the human footprint index (Mu et al. 2022), hunter activity, and corn productivity. We created a spatial representation of corn productivity by multiplying the county‐level yield for 2018 (converted to bushels per 900 m^2^) by the United States Department of Agriculture Cropland Data Layer for corn (USDA 2023). We used hunter activity as a metric to represent the potential availability of intentional bait on the landscape by multiplying the density of roads, the proportion of public land, and the number of harvested bears using bait within each sub‐county game management unit within our home range buffer of sample locations. Hunter harvest surveys by the Wisconsin Department of Natural Resources determined the number of harvested bears using bait and identified these contributing factors to the increasing accessibility to bear hunters and consequently the amount of bait on the landscape.

### 2.3 Disentangling Intentional and Unintentional Human Food Subsidies

We estimated proportional diets of black bears with a Bayesian isotopic mixing model in the R (R Core Team 2024) package *MixSIAR* (Stock et al. 2018). We first identified isotopically distinct dietary groups using a K nearest‐neighbor randomization test (Figure S1; Rosing et al. 1998). To understand the use of human food subsidies broadly and disentangle specific sources of human food subsidies, we used isotopically distinct groups of animal matter, hard mast, intentional subsidies (bait), and vegetation/soft mast, but combined corn and food waste into a single source group to represent unintentional subsidies as sources in the mixing model (Figure S1). We estimated proportional diet by averaging the δ^13^C and δ^15^N values for black bear hair samples that occurred at the same location (*n* = 183; Figure 1); thus, our diet estimates represent averaged black bear diet at each sample location. We applied tissue‐specific trophic discrimination factors for omnivores that consume a mixed diet, such as black bears that forage on local vegetation and human food subsidies from C3 and C4 photosynthetic pathways (Δ^13^C = 2 [SD = 0.2]; Δ^15^N = 3.3 [SD = 0.2]; Stephens et al. 2023). The estimation and application of discrimination factors for diet reconstruction have been a major area of research and numerous approaches have been advocated (Hopkins et al. 2021; Stephens et al. 2023). Consequently, we also ran an alternate mixing model that applied source‐specific trophic discrimination factors that varied by diet item (Table S2; Hopkins et al. 2021; Kirby et al. 2017). Given that black bears do not assimilate prey keratin (hair), we adjusted the TDF of δ^13^C for samples of deer hair to account for offsets between the isotopic discrimination of hair and muscle tissue (δ^13^C = 1.3; Stephens et al. 2022). Models included concentration dependencies for each diet group from mean digestible elemental concentrations (Hopkins and Ferguson 2012; Kirby et al. 2017). We estimated proportional dietary inputs using an uninformative prior and ran 3 chains of 300,000 iterations, removed the first 200,000 iterations as burn‐in, and then thinned posterior samples to every 100th sample (Stock et al. 2018). We assessed model convergence with trace plots and Gelman‐Rubin statistics (Brooks and Gelman 1998; Gelman and Rubin 1992). We modeled the change in proportional diets across single predictor models (*n* = 7) including the proportion, connectivity, and edge density of natural land cover, cumulative productivity, human footprint index, hunter activity, corn productivity, and a null model. All variables were scaled and centered prior to model fitting.

### 2.4 Human Subsidy Landscape

We projected a diet landscape using relationships between the proportion of unintentional and intentional subsidies in black bear diet and landscape variables fitted with our Bayesian mixing models. For each predictor, we used a moving window, approximately equal to the average home range, and calculated model predictors within each raster cell. We predicted the proportion of human subsidies across the whole spatial domain using raster surfaces of our landscape variables as predictors. We aggregated rasters to match the lowest resolution among all predictors (500 m). We averaged all predicted univariate human subsidy landscapes into a single estimate. To explore black bear diet in areas where they are less common, we allowed our predictions to be extrapolated beyond the modeled predictor values but denoted areas outside of the range of observed values from sample locations (Table S3 and Figure S2).

We used Bayesian generalized linear models to test the effect of the consumption of human food subsidies on the number of reported agricultural and conflict complaints from black bears. We obtained the number of reported complaints in 2018 by Wisconsin county from the Cooperative Bear Damage Management Program (USDA‐WS 2018). We calculated the mean proportion of human food subsidies for each county from our projected human subsidy landscape. We also controlled for two additional covariates, likely to influence the number of reported complaints, by including the relative abundance of black bears by county from the number of harvested bears (Wisconsin Department of Natural Resources) and human population density by county (Wisconsin Department of Health Services). We implemented a negative binomial regression model in the R packages *brms* (Bürkner 2017) and ran four chains for 4000 iterations and discarded the first 2000 iterations as warmup. To evaluate model convergence, we required *R hat* values < 1.01 and visually inspected traceplots (Gelman and Rubin 1992). To describe the posterior effects of parameter estimates, we calculated the 95% highest density interval (HDI) and probability of direction (pd) using the R package *bayestestR* (Makowski et al. 2019). The probability of direction represents whether the posterior distribution of the parameter has an effect in a particular direction (i.e., positive or negative).

## 3 Results

**FIGURE 2:**
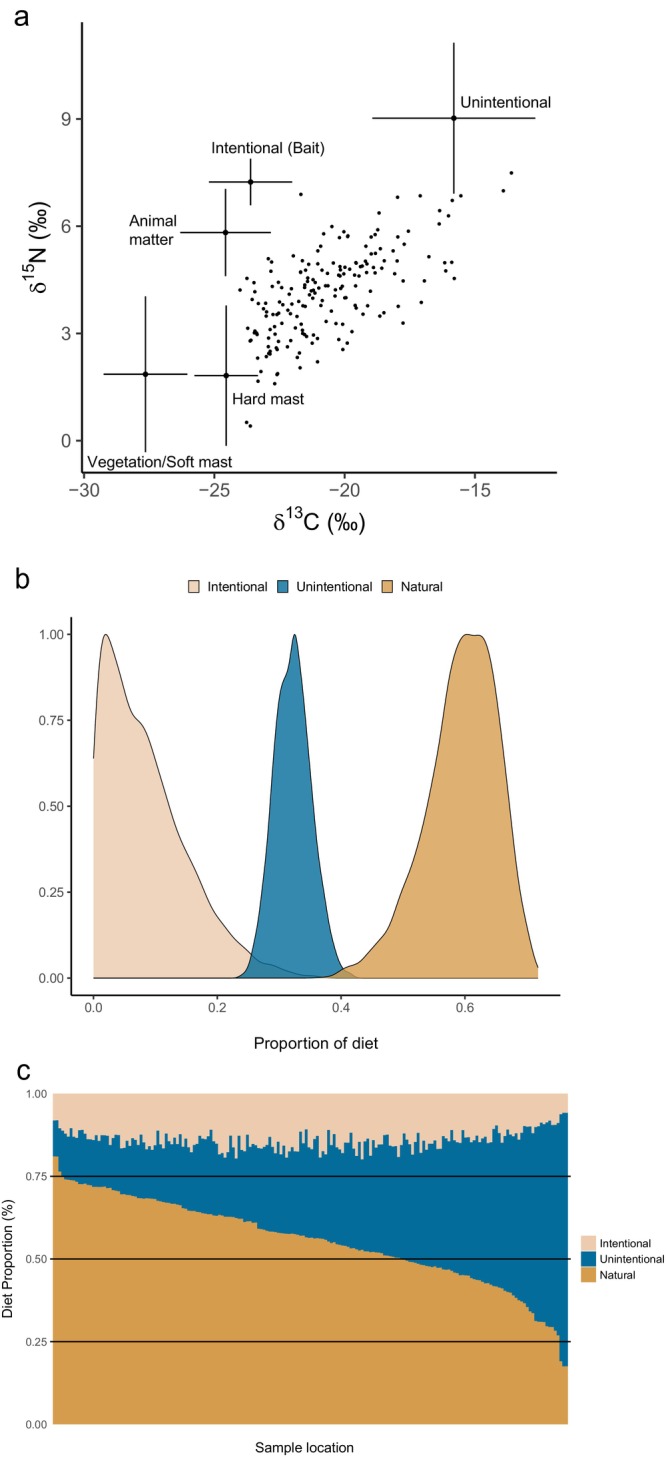
Mean carbon (δ^13^C) and nitrogen (δ^15^N) isotope value and standard deviation of dietary source groups including American black bear values (black points). Trophic discrimination factors have been applied to dietary sources (Δ^13^C = 2; Δ^15^N = 3.3; Stephens et al. 2023). Population diet of American black bears ( *Ursus americanus* ) in Wisconsin estimated from a Bayesian isotopic mixing model (b), and proportional diet estimate for each sample location (c). We estimated three functional dietary groups that included: Natural forage (soft mast, hard mast, herbaceous plants, and animal matter [deer and ants]), unintentional human food subsidies (corn and human food), and intentional human food subsidies (bait).

**FIGURE 3:**
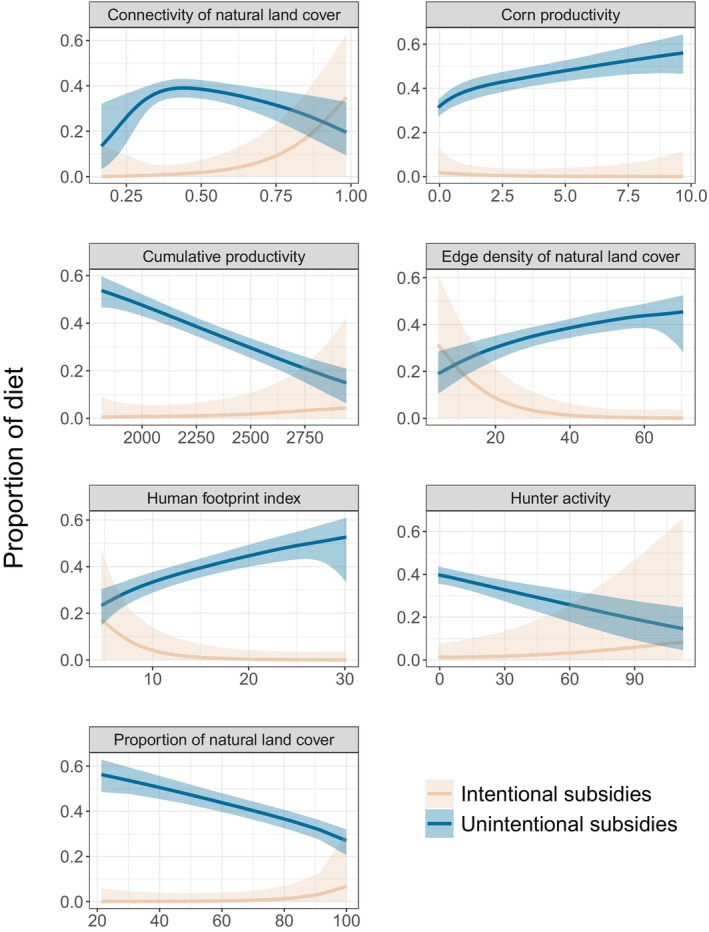
Functional relationships between the consumption of intentional and unintentional human food subsidies and landscape variables for American black bears ( *Ursus americanus* ) in Wisconsin fitted using a Bayesian isotopic mixing model. Median estimates of proportional diet with 95% Bayesian credible intervals. Unintentional subsidies included corn and food waste, and intentional human food subsidies included bait used during hunting.

Averaging across all sample locations, black bears consumed mostly natural foods (60%; 95% CI [46%, 69%]) compared to intentional (7%; 95% CI [0%, 25%]) and unintentional (32%; 95% CI [27%, 38%]) human subsidies (Figure 2a). Nevertheless, we found substantial contributions of human foods, with 39% of sample locations having minimal (15%–30%) consumption of intentional subsidies while 44% of sample locations showed a moderate (30%–50%) or high (> 50%) consumption of unintentional subsidies (Figure 2b). Increasing vegetation productivity (i.e., cumulative productivity) and proportion of natural land cover resulted in a declining proportion of human food subsidies (Figure 3). Conversely, the consumption of unintentional subsidies was positively associated with corn productivity, edge density of natural land cover, and human footprint index (Figure 3). We found intentional subsidies were likely to increase with hunter activity and connectivity of natural land cover (Figure 3).

**FIGURE 4:**
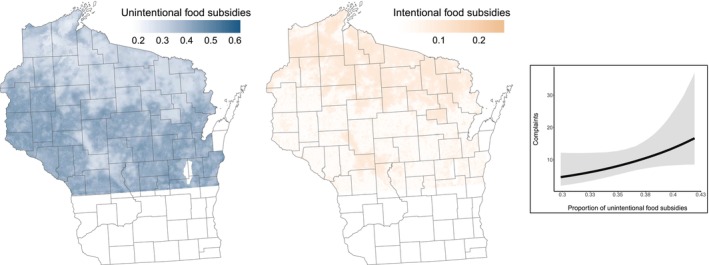
Projected diet landscapes of intentional and unintentional human food subsidies of American black bears ( *Ursus americanus* ) in Wisconsin from Bayesian isotopic mixing models. Bayesian generalized linear model prediction for the effect of consumption of unintentional human food subsidies on the number of reported complaints from black bears in Wisconsin. Intentional human food subsidies included bait used during hunting activities, and unintentional subsidies included corn and human food.

Our projected human subsidy landscape followed a latitudinal trend that followed corn production and natural land cover. The consumption of intentional subsidies was highest and more continuous through portions of northern and central Wisconsin that feature more connected public land and where hunter activity is highest (Figure 4). However, the projected consumption of unintentional subsidies was greatest in the southern and western portions of the state (Figure 4). We found that the increasing consumption of unintentional, but not intentional subsidies corresponded with more reported complaints (Figure 4; Table 1). The posterior distribution for the effect of the consumption of unintentional subsidies had a 94% positive probability of direction, and 0.5% occurred inside the region of practical equivalence.

**TABLE 1:** Parameter estimates from the Bayesian negative binomial regression model explaining the county level number of black bear complaints as a function of the proportion of human food subsidies, relative abundance of black bears, and human population density in Wisconsin.

Parameter	Median	95% CI	Pd	Rhat	ESS
Intercept	−1.67	−5.58, 2.03	86.9%	1.00	5319
Unintentional subsidies	10.53	−1.44, 22.73	95.9%	1.00	5524
Black bear abundance	0.01	0.01, 0.02	100%	1.00	5848
Human population density	0.00	−0.01, 0.01	70.7%	1.00	7612

Abbreviations: CI, credible interval; ESS, effective sample size; pd, probability of direction.

## 4 Discussion

Black bears consumed a substantial proportion of human food subsidies, but landscape composition, configuration, and productivity were correlated to differences in the consumption of intentional and unintentional sources. This led to a spatially heterogeneous human subsidy landscape and highlighted important differences in the drivers of the consumption of intentional and unintentional subsidies. Particularly, the consumption of intentional subsidies increased in areas with connected public lands and higher hunter activity, while the consumption of unintentional subsidies increased with corn productivity in disjunct areas of natural land cover. By disentangling these two sources of human food subsidies and quantifying consumption based on landscape features, we predicted more conflict with people and property in areas with greater consumption of unintentional subsidies. Consequently, mapping the human subsidy landscape provides areas where management actions to reduce the availability of intentional and unintentional subsidies may be most beneficial and predicts areas to promote best practices to mediate human‐wildlife interactions and conflict.

Many sampling locations showed substantial consumption of human‐related foods. Our estimates are similar to previously reported consumption in other highly food‐conditioned populations of black bears (Hopkins et al. 2014) and globally from other Ursids in human‐dominated landscapes (Kavčič et al. 2015; Sato et al. 2004; Vulla et al. 2009). Our diet estimates from hair largely represented consumption during the previous summer and fall, including the period of hyperphagia when bears tend to increase the consumption of human food subsidies to meet physiological demands (Baruch‐Mordo et al. 2014; Johnson et al. 2015). Black bear diets often track seasonal availability and can mitigate shortages in natural foods through the consumption of human food subsidies. While we lacked the availability of natural foods like hard mast (oak, hazel, and dogwood) in Wisconsin for 2018, regional indices for neighboring Minnesota reported an average to above‐average year. Consequently, our diet estimates likely represent an average year and not a year in which mast scarcity would lead to a diet switch. Additionally, evidence of diet switching from natural forage remains inconsistent, especially in human‐modified landscapes (Ditmer et al. 2016; Merkle et al. 2013). Black bears have also not shown a preference for selecting natural forage over human food subsidies when equally available (Kirby et al. 2017). Some degree of specialization on human food subsidies would be predicted regardless of natural forage availability due to the spatial and temporal regularity of human food subsidies and the propensity of learned behaviors in black bears (Mazur and Seher 2008).

We found support for landscape variables that influenced different consumption of human food subsidies and contrasting relationships between unintentional and intentional human subsidies. Regional differences in consumption varied substantially across our sampling locations. Bears in the southern and western portions of the state consumed large amounts of unintentional subsidies, which, in some cases, represented the majority of their diet, largely driven by corn production and disjunct areas of natural land cover. Our analysis revealed that black bears will readily consume unintentional subsidies when the landscape no longer supports natural forage as vegetation productivity, natural land cover, and connectivity decrease. Given the high caloric content of crops can increase weight gain in black bears (Ditmer et al. 2016), it has been postulated that the addition of human food subsidies has likely contributed to a large (estimated at ~28,000) and growing (3.4% annually since 1988) black bear population in Wisconsin (Kirby et al. 2017). Black bear densities in Wisconsin are estimated to be 2× higher than the neighboring states of Minnesota and Michigan. While recent corn production exceeds that of Michigan, Minnesota produces double that of Wisconsin, and even in agricultural landscapes, showed mixed use of crops (Ditmer et al. 2016). However, Wisconsin has the most liberal baiting regulations in the region, and the combination of crops and bait may mediate any shortages in natural forage with high caloric and predictable food and, thus, contribute to the high population densities.

The extensive consumption of human food subsidies can increase fecundity, body mass, and shorten hibernation periods (Beckmann and Berger 2003b; Johnson et al. 2018; Kirby et al. 2019) suggesting important additive effects on food availability. However, despite the potential physiological and reproductive benefits, the use of human‐modified landscapes can elicit greater stress (Babic et al. 2023; Ditmer et al. 2018; Kirby et al. 2019; Støen et al. 2015) and lead to increased conflict with humans that can reduce the survival of adults and cubs (Johnson et al. 2020; Wynn‐Grant et al. 2018). Indeed, we found the consumption of unintentional subsidies was indicative of more reported complaints and conflict, after accounting for variation in relative black bear abundance and human densities. Black bears have increased their use of human‐modified landscapes across much of their range (Hristienko and McDonald 2007) and minimizing the accessibility of unintentional human food subsidies will be required across more rural communities and peri‐urban systems to reduce conflict. Consequently, predicting diet and landscape drivers that lead to increased human‐wildlife conflict provides spatially explicit areas for managers to apply conflict avoidance strategies (e.g., removal of bear attractants, increase awareness programs) and targets for habitat management.

The ubiquity of human food subsidies and their ability to fundamentally alter individuals, populations, and communities requires the consideration of their impact throughout the food webs, even when human food subsidies are not directly consumed (Manlick and Pauli 2020). Ecologists and resource managers have multiple tools to estimate diet, but the easy digestibility, diversity, and processing of human‐derived foods can obscure their quantification and importance with alternate methods (i.e., mechanical sorting and metabarcoding) (e.g., Lacin Alas et al. 2025). Enrichment in carbon has had widespread use as a tracer of human‐derived foods in temperate North American systems due to differences between corn products and native vegetation (Hopkins et al. 2014; Kirby et al. 2016; Newsome et al. 2015). This categorization often works well; however, intentional food subsidies can include a combination of food products that are isotopically similar to native and corn‐derived products. In our system, we found intentional food subsidies to be isotopically distinct from both natural forage and unintentional food subsidies, and we detected little contribution of intentional food subsidies at this regional scale (≤ 20% across all locations). While the isotope values and variation of intentional food subsidies were estimated directly from 42 baiting stations, there are a few notable limitations. First, despite our observed variation in the isotope values of intentional foods, the contents of bait stations are often derived from commercial agricultural products and unlikely to resemble natural vegetation in δ^15^N values. Indeed, we found intentional subsidies to be enriched in δ^15^N and only slightly enriched in δ^13^C compared to natural vegetation. The isotope values of intentional subsidies trended towards animal matter, and additional techniques could improve this differentiation (e.g., compound specific stable isotope analysis from amino acids or fatty acids; Twining et al. 2020). If products derived from corn (e.g., C4) were underrepresented in our intentional subsidies, we would have underestimated their contribution and overestimated unintentional subsidies. We have overall confidence in our representation of these sources as they spatially track land cover variables that we would expect to predict intentional and unintentional human subsidies. Although complimentary, emerging and the development of novel approaches could further disentangle the consumption of intentional subsidies and are necessary to better assess the impact of management regulations and policy on wildlife populations. The isotopic separation of dietary groups is also influenced by the selection of trophic discrimination factors. We applied two sets of trophic discrimination factors that account for differences in diet sources and found minimal change in population‐level estimates (≤ 7%), but our conclusions and spatial patterns remained consistent.

The variation in the human subsidy landscape and consumption of intentional and unintentional subsidies highlights the need to disentangle these two different pathways when managing for the impact of human food subsidies on this large carnivore. Our results suggest that human food subsidies contribute a substantial proportion of the bear diet, especially along their expanding range into human‐dominated landscapes and can ultimately lead to greater conflict with people and property. Predicting the consumption of human food subsidies provides areas for resource managers to monitor and targets for prescriptions that improve natural land cover or reduce the availability of human food subsidies. Human‐wildlife interactions are fundamental to the conservation and management of wildlife but pose challenges, as outcomes are often conflicting with simultaneous goals to enhance (e.g., increased hunting opportunities, recovery of endangered species) or reduce (e.g., agricultural and property damage) interactions. Resource management, thus, needs to assess the relationships between foraging ecology and the composition, configuration, and productivity of human‐modified landscapes to be able to anticipate use and the potential for human‐wildlife interactions and conflict.

## Author Contributions

**Matthew M. Smith:** conceptualization (equal), data curation (equal), formal analysis (lead), investigation (equal), methodology (lead), writing – original draft (lead), writing – review and editing (lead). **David M. MacFarland:** conceptualization (equal), data curation (equal), funding acquisition (equal), investigation (supporting), writing – review and editing (supporting). **Jennifer L. Price Tack:** conceptualization (equal), data curation (equal), funding acquisition (equal), investigation (equal), writing – review and editing (equal). **Jonathan N. Pauli:** conceptualization (equal), funding acquisition (equal), methodology (supporting), supervision (lead), writing – review and editing (equal).

## Conflicts of Interest

The authors declare no conflicts of interest.

## Supporting information


Data S1.

## Data Availability

Data and code that supports the findings of this study are available in Figshare at 10.6084/m9.figshare.29504786.
