# Supplementary material for: The Intentional and Unintentional Human Food Subsidy Landscape for a Large Carnivore
Source: Ecol Evol. 2025 Aug 21;15(8):e71853. doi: 10.1002/ece3.71853 (PMC12368351; doi:10.1002/ece3.71853)

**Supporting Information**

***The intentional and unintentional human food subsidy landscape for a large carnivore***

| Table S1: Mean, standard deviation, and concentrations of δ^15^N and δ^13^C isotopic values of diet items consumed by American black bears in Wisconsin. Values were used in isotopic mixing model to quantify proportional diets. | | | | | | | |
| --- | --- | --- | --- | --- | --- | --- | --- |
| Sources | Meand13C | SDd13C | Meand15N | SDd15N | Concd13C | Concd15N | n |
| Animal matter | -26.57 | 1.74 | 2.52 | 1.22 | 0.52 | 0.12 | 52 |
| Bait | -25.61 | 1.60 | 3.94 | 0.65 | 0.51 | 0.01 | 27 |
| Unintentional | -17.81 | 3.13 | 5.72 | 2.11 | 0.50 | 0.05 | 113 |
| Hard mast | -26.55 | 1.23 | -1.48 | 1.96 | 0.47 | 0.04 | 33 |
| Vegetation/Soft mast | -29.64 | 1.61 | -1.44 | 2.18 | 0.47 | 0.04 | 110 |

| Table S2: Alternate set of trophic discrimination factors used in Bayesian mixing models to estimate proportional diets of American black bears in Wisconsin (Hopkins et al., 2021; Kirby et al., 2017). | | | | |
| --- | --- | --- | --- | --- |
| Sources | Meand13C | SDd13C | Meand15N | SDd15N |
| Animal matter | 2.1 | 0.1 | 3.9 | 0.3 |
| Bait | 4.1 | 0.3 | 2.8 | 0.2 |
| Unintentional | 1.4 | 0.5 | 2.4 | 0.2 |
| Hard mast | 3.4 | 0.2 | 2.4 | 0.2 |
| Vegetation/Soft mast | 3.4 | 0.2 | 2.4 | 0.2 |

| Table S3: Mean, standard deviation (SD), and range of variables from sampling locations used to model diet of American black bears in Wisconsin. | | | |
| --- | --- | --- | --- |
|  | Mean | SD | Range |
| Connectivity of natural land cover | 0.51 | 0.14 | 0.17, 0.99 |
| Cumulative productivity | 2293 | 209 | 1809, 2936 |
| Proportion of natural land cover | 78.1 | 21.8 | 21.8, 99.3 |
| Human footprint index | 13.0 | 5.5 | 4.8, 30.1 |
| Corn productivity | 1.7 | 2.4 | 0, 9.6 |
| Edge density of natural land cover | 35.3 | 14.6 | 4.8, 70.1 |
| Hunter activity | 2405 | 1728 | 52, 9540 |

**Figure S1**: Mean carbon (δ13C) and nitrogen (δ15N) isotope value and standard deviation of dietary source groups including American black bear values (black points). Trophic discrimination factors in Table S2 have been applied to dietary sources. Population diet of American black bears (Ursus americanus) in Wisconsin estimated from a Bayesian isotopic mixing model (b), and proportional diet estimate for each sample location (c). We estimated three functional dietary groups that included: Natural forage (soft mast, hard mast, herbaceous plants, and animal matter [deer and ants]), unintentional human food subsidies (corn and human food waste), and intentional human food subsidies (bait).

**Figure S2**: Functional relationships between the consumption of intentional and unintentional human food subsidies and landscape variables for American black bears (Ursus americanus) in Wisconsin fitted using a Bayesian isotopic mixing model. Median estimates of proportional diet with 95% Bayesian credible intervals. Unintentional subsidies included corn and food waste, and intentional human food subsidies included bait used during hunting.

**Figure S3**: Projected diet landscapes of intentional and unintentional human food subsidies of American black bears (Ursus americanus) in Wisconsin from Bayesian isotopic mixing models. Bayesian generalized linear model prediction for the effect of consumption of unintentional human food subsidies on the number of reported complaints from black bears in Wisconsin. Intentional human food subsidies included bait used during hunting activities, and unintentional subsidies included corn and human food**.**

**Figure S4**: Projected diet landscapes of intentional and unintentional human food subsidies of American black bears (Ursus americanus) in Wisconsin from Bayesian isotopic mixing models. Bayesian generalized linear model prediction for the effect of consumption of unintentional human food subsidies on the number of reported complaints from black bears in Wisconsin. Intentional human food subsidies included bait used during hunting activities, and unintentional subsidies included corn and human food waste. Grey polygons depict areas that are extrapolated outside of observed values.


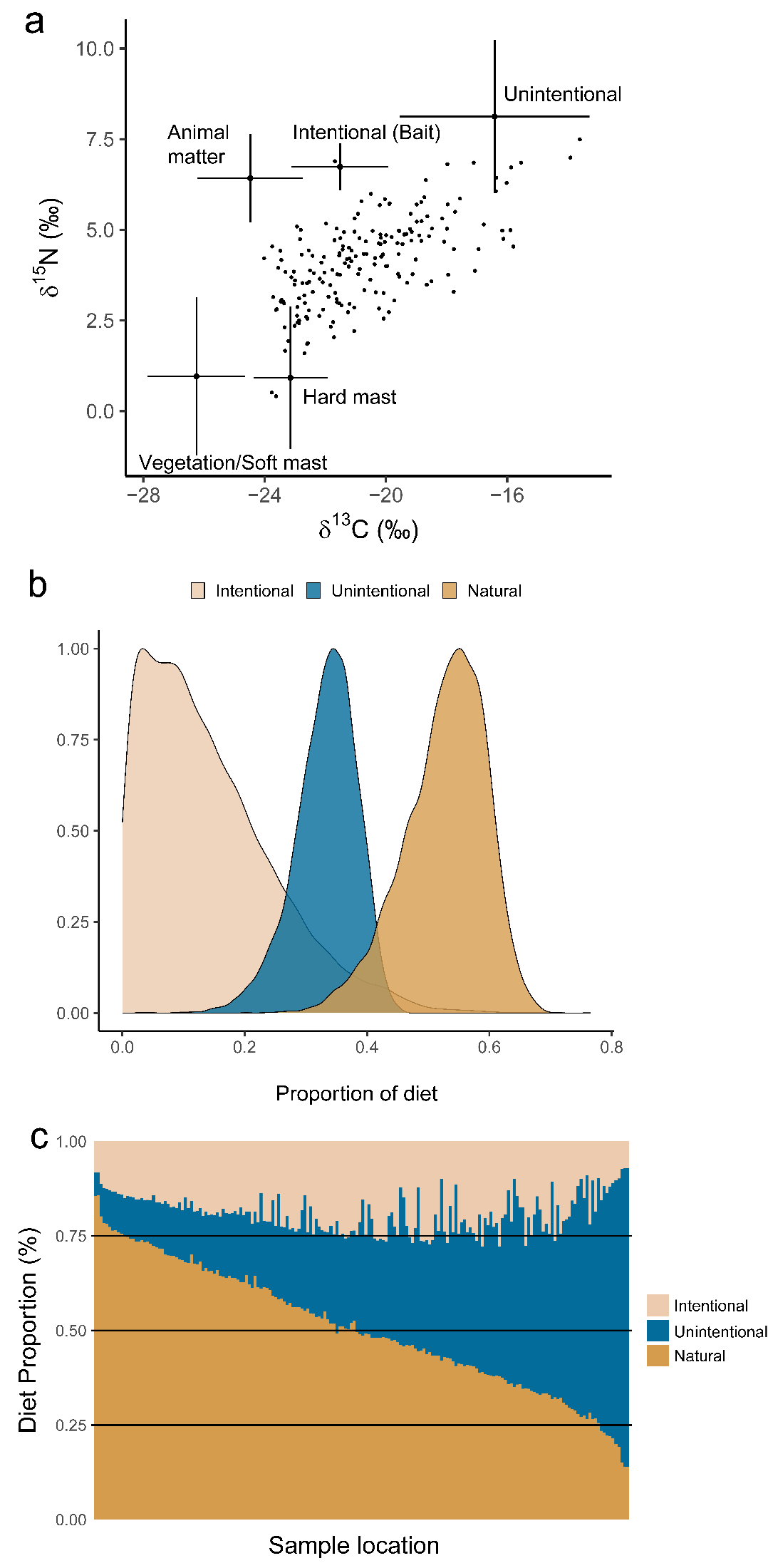


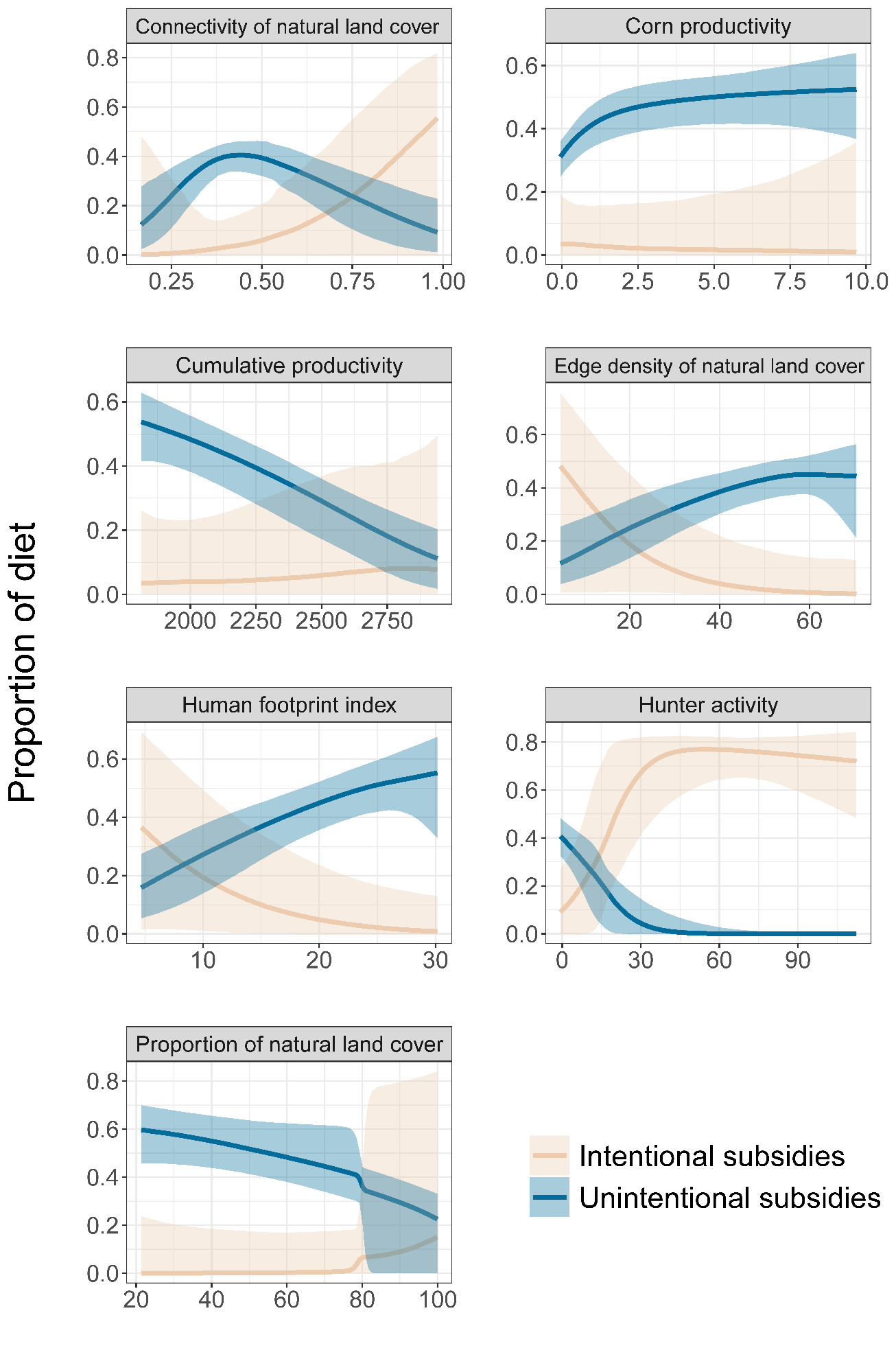


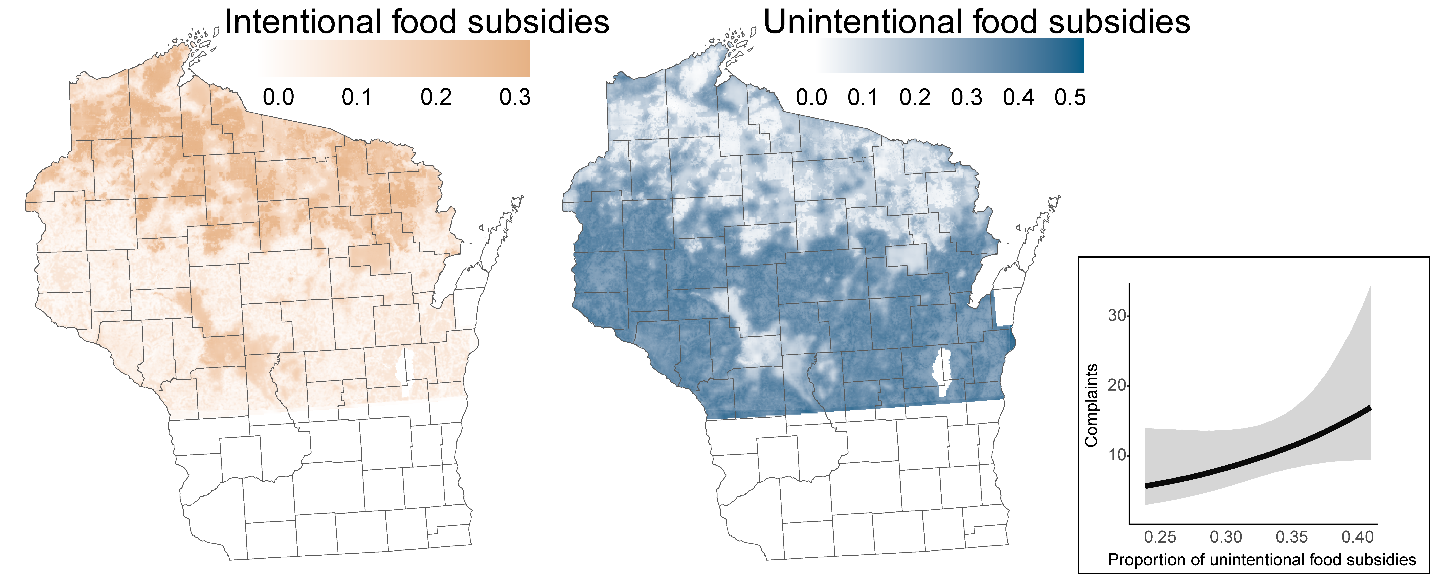


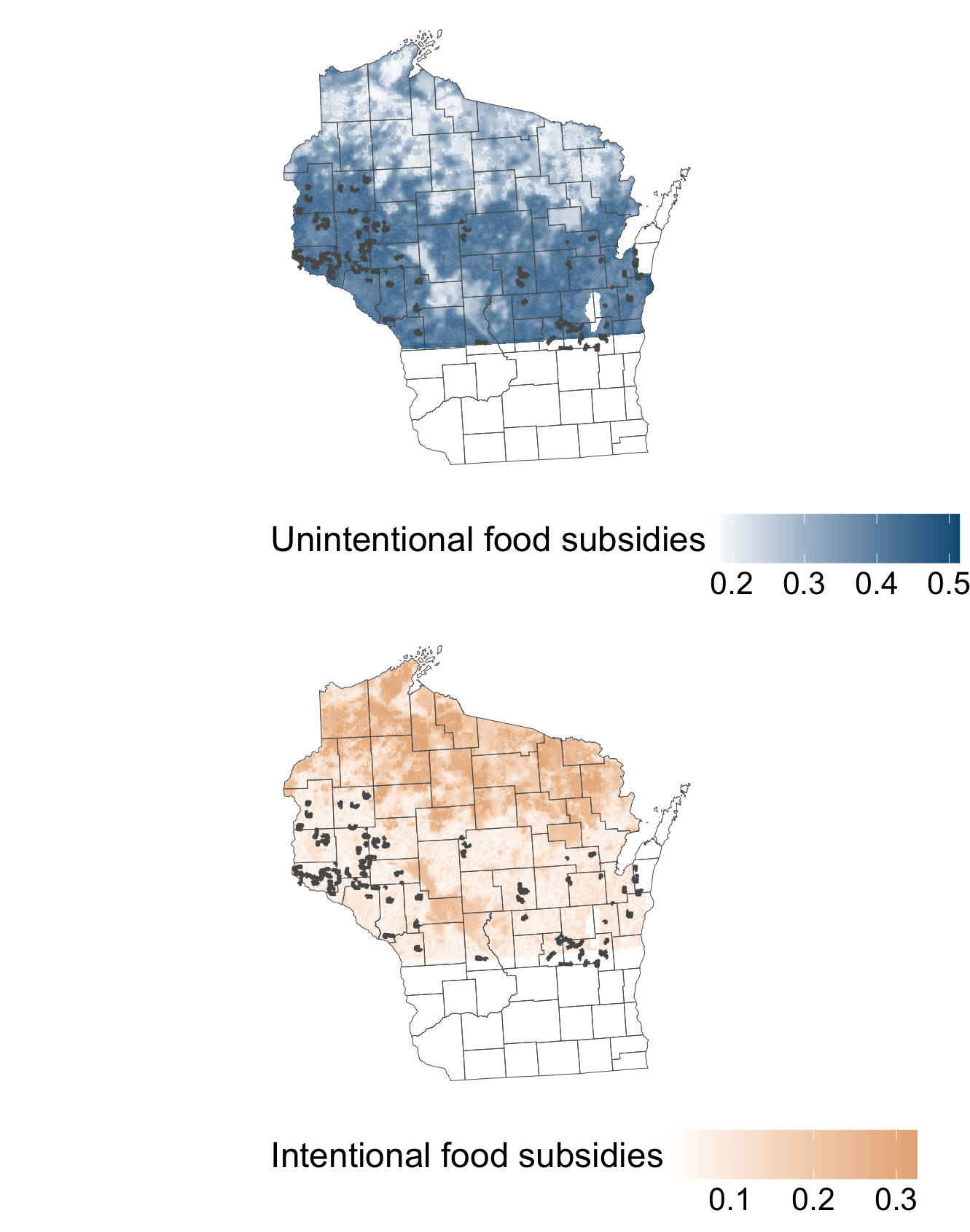

Supplement: Supplementary file 1 — Data S1. [file ECE3-15-e71853-s001.docx]
